# Dyslexia: the Role of Vision and Visual Attention

**DOI:** 10.1007/s40474-014-0030-6

**Published:** 2014-09-27

**Authors:** John Stein

**Affiliations:** Department of Physiology, Anatomy & Genetics, University of Oxford, Sherrington Building, Parks Road, Oxford, OX1 3PT UK

**Keywords:** Visual dyslexia, Magnocellular neurons, Dorsal visuomotor pathway, Visual attention, Coloured filters, Omega-3s, Genetic basis

## Abstract

Dyslexia is more than just difficulty with translating letters into sounds. Many dyslexics have problems with clearly seeing letters and their order. These difficulties may be caused by abnormal development of their visual “magnocellular” (M) nerve cells; these mediate the ability to rapidly identify letters and their order because they control visual guidance of attention and of eye fixations. Evidence for M cell impairment has been demonstrated at all levels of the visual system: in the retina, in the lateral geniculate nucleus, in the primary visual cortex and throughout the dorsal visuomotor “where” pathway forward from the visual cortex to the posterior parietal and prefrontal cortices. This abnormality destabilises visual perception; hence, its severity in individuals correlates with their reading deficit. Treatments that facilitate M function, such as viewing text through yellow or blue filters, can greatly increase reading progress in children with visual reading problems. M weakness may be caused by genetic vulnerability, which can disturb orderly migration of cortical neurones during development or possibly reduce uptake of omega-3 fatty acids, which are usually obtained from fish oils in the diet. For example, M cell membranes require replenishment of the omega-3 docosahexaenoic acid to maintain their rapid responses. Hence, supplementing some dyslexics’ diets with DHA can greatly improve their M function and their reading.

## Introduction

Recently, Durham Professor of Education, Joe Elliot, and Russian geneticist, Elena Grigorenko, published a highly controversial book, *The Dyslexia Debate* [1], in which they argue that the concept of “dyslexia” should be abandoned. First, they claim that there is insufficient agreement even among experts about what dyslexia really is for there to be consistent diagnostic criteria, so you can “shop around” for the diagnosis, which then confers on you unfair resources, such as computer aids and exam concessions. Second, they suggest that making the diagnosis makes no difference to how you are treated; since both dyslexics and other poor readers benefit from structured phonological treatment, there is no point in making the diagnosis. In short, they think that there is no fundamental difference between dyslexics and any other poor readers. Instead, lavish resources ought to be available to all. All of these points are true to some extent, but they still do not render the concept useless.

The main problem with their analysis is that they assume throughout that all reading problems are primarily due to phonological difficulties—inability to segment word sounds into their constituent phonemes to match them with the letters that stand for them. But this explanation is actually more of a tautology; the very essence of reading is translating visual symbols into the sounds they stand for, so calling it a phonological problem merely redescribes the symptoms. Furthermore, it entirely ignores the visual and the many other non-phonological problems that dyslexic children commonly encounter [2].

Although there has been an attempt to shift away from the “discrepancy” approach to diagnosis [3], among practitioners the diagnosis of dyslexia still depends on showing that a person’s phonological, reading and spelling skills are well below what one would expect for their age and other aspects of their basic intelligence. But dyslexia is actually much deeper than this. It is better thought of as a neurological “syndrome” involving much more than just reading [4]. Clinically, a clear pattern of symptoms and signs signify dyslexia, including a strong genetic predisposition, brain differences (which have been clearly demonstrated by both structural and functional imaging) and slow visual processing and auditory processing (which probably underlie the visual and phonological reading problems that are seen), together with difficulties in focusing attention, poor sequencing, poor timing, left–right confusions and poor short-term memory. This pattern is so distinct from other causes of poor reading that it can be easily diagnosed, and, contrary to the views of Elliot and Grigorenko, diagnosis is useful because it can lead to more effective treatments.

## Visual Contributions to Reading

Here, I am going to concentrate on visual aspects of dyslexia. Reading draws heavily on visual processing; it is glaringly obvious that letters have to be seen and identified and put in the right order in order to be read properly. Even in practised readers, these visual processes remain essential and are rate limiting [5]. But about 5 % of all children and about half of all dyslexic children complain of visual problems when they try to read: letters appear to blur, move around and go double, so the children cannot see them properly, which often gives them eyestrain and headaches [6]. Obviously, such symptoms interfere with learning to read.

## Visual Perceptual Stability

What causes these visual symptoms? Answering this question demands a brief digression into how we keep our visual world stationary even though our eyes, and hence retinal images, are moving all the time. Before each eye movement, the brain—in particular, the cerebellum [7]—automatically predicts where the images are going to end up. Then feedback from the retina signals how the images have moved, and eye muscle stretch receptors signal the new position of the eyes. These consequentially precisely measured image movements are then subtracted from our perception, and so we see no apparent movement of the world, and the world remains satisfactorily stationary.

However, if eye movements are not predicted and compensated for in this way, then objects do appear to move. If you push your eyeball from side to side, that movement is not predicted, and even though consciously you know you have caused it, the world appears to move around. Thus, accurately predicting where the eyes will move is essential. Alcohol particularly disrupts the cerebellum; hence, too much alcohol prevents proper prediction; the image movements following eye movements are not compensated for properly, and this makes the world appear to stagger around.

This predictive process needs appropriate signals of upcoming movements and of their progress in order for you to accurately compute by how much the images will move, so that that their effects can be accurately subtracted from conscious perception. This process starts with redirecting attention. Before you move your eyes, your attention moves to focus on the next target of your gaze, and this provides the first information about the metrics of the forthcoming eye movement. This attentional change can either be driven by a powerful “bottom-up” visual signal appearing suddenly (e.g. a fly alighting on the page) or “top-down” you make a decision to move to view a new target (e.g. during reading, to move to the next word the moment you have analysed the last word).

In addition to control of the focus of attention, the visual system keeps the visual world stable, despite eye movements, in at least two other important ways. The first is by compensating for miniature eye movements. Even during fixation on a letter or word, the eyes make small movements—microsaccades and drifts [8]. These are actually vital, because the retinal pigments that transduce light into electrical nerve impulses are bleached in the process and thereafter cannot signal again for a second or so, so small movements are required to bring new pigment molecules into play. If all eye movements are prevented, then vision fades completely. These small eye movements do not cause blur, however, because they are compensated for by the visual system. Movement-sensitive ganglion cells signal the jitter caused by the small movements to the visual cortex. Since this is identical all over the retina because the whole eye is moving, this baseline level of motion can be estimated and simply discounted computationally. This was shown by an ingenious set of “jitter” experiments by Murakami and Cavanagh [9].

Larger, unwanted eye movements during fixations are also efficiently limited by the visual system. Such movements cause large image movements on the retina. These are fed back to the eye muscle control system, which forms a negative feedback servomechanism that causes the eyes to move back by the same amount as the images have moved, thus locking the eyes on the target.

## The Visual Magnocellular System

These motion-sensing and attentional processes depend very much on the properties of the “visual magnocellular system”. The “ganglion” cells in the retina are the neurons that send information from the eye to the rest of the brain—in particular, to the visual cortex, which is situated on the back of the occipital cortex at the very back of the brain. They gather signals from the light receptors at the back of the eye and project them back to the brain. Ten percent of them are much larger than the others; hence, they are called magnocellular (M) neurones [10]. They are highly specialised for timing visual events. They capture information over a very large retinal area—about a square millimetre; this is up to 50 times larger than that of the small parvocellular (P) ganglion cells. Although the latter are smaller, they are 10 times more numerous. Being smaller, they respond more slowly than M cells, but they can define the fine detail and colour of objects. Therefore, for reading, it is actually the P system that provides the main input into the brain’s letterbox—or the “visual word form area” (VWFA)—where letters are identified.

The magnocellular neurones cannot define letters in such fine detail, and they do not discriminate between colours; but because they are larger, they respond and conduct signals much more rapidly than the parvo cells. This means that they are much more sensitive to temporal changes in the outside world, such as flicker and movement. Thus, they can rapidly signal changes in the environment, and this is particularly important for capturing attention; they provide the main signals for visual guidance of attention and of eye and limb movements. In particular, they direct the P system to each letter in order to identify it and its position in a word [11].

Both the M and P ganglion cells project to the lateral geniculate nucleus (LGN) of the thalamus en route to the primary visual (striate-V1) cortex, which is situated at the back of the occipital lobe at the back of the brain. But the M cells project to the magnocellular layers of the LGN, whereas the P cells project to the parvocellular layers. This separation is preserved in the onwards projection from the LGN via the optic radiations to layer 4 of the striate cortex. Here, magnocells project to layer 4C alpha and parvo cells project to layer 4C beta. Thereafter, however, M and P inputs become intermixed.

## Dorsal and Ventral Visual Pathways

There are two major forward projections from the primary visual cortex to the rest of the brain: the dorsal and ventral pathways [12]. The dorsal “where” (and “when”) pathway mediates the visual guidance of attention and of eye and limb movements, and its main visual input is provided by the magnocellular system. In contrast, the slower ventral “what” pathway passes forward ventrally underneath the occipitotemporal cortex. Its main function is to detect what the texture, form and colour of objects are, in order to identify them; hence, the VWFA lies within this pathway [13].

The dorsal route passes dorsally to the visual motion-sensitive area (MT/V5), which is situated in the middle temporal gyrus at the occipitotemporal junction, and thence to the posterior parietal cortical (PPC) angular and supramarginal gyri. In the left hemisphere, these parietal areas are particularly important for reading. Dejerine [14] and Geschwind [15] thought they were responsible for associating the visual form of a word with its sound and meaning. But we now know that the VWFA does the visual part of this, and it is located in the fusiform gyrus, which lies in the ventral, P-dominated, form-analysing pathway [13].

The job of the angular and supramarginal gyri is now thought to be to focus visual attention very rapidly on the letters and their order in words to be read. The ventral route and VWFA system can recognise individual letters, but they cannot code their precise location, i.e. their order in a word—which, of course, is vitally important for reading. So the rapid dorsal route angular and supramarginal gyri send back to V1 and to the VWFA a signal about where to attend in order to identify letters; thereby, it specifies their order in a word [16]. Thus, when one is learning to read, the angular and supramarginal gyri seem to help the VWFA to focus on individual letters in order to identify them and their order, then these are linked with the word sounds stored in Wernicke’s area, which is situated at the back of the superior temporal gyrus [17]. But if the word is already in the reader’s visual lexicon, its meaning can be rapidly grasped by direct connection of the VWFA via the arcuate fasciculus to Broca’s speech area in the inferior frontal gyrus, for Broca’s area supplies the meaning of words even if they are not actually spoken [18].

## The Magnocellular Cell Lineage

From the supramarginal and angular gyri, the main target of the dorsal pathway is the inferior frontal gyrus (Broca’s area) and prefrontal cortex, where conscious decisions for behaviour are made. The nerve cells throughout the whole of the dorsal pathway tend to be larger than those in the ventral pathway, and, like retino-geniculate M cells, they appear to derive from the same developmental lineage, because they all express a particular set of surface signature antigens that are recognised by selective antibodies such as CAT-301 [19]. Every cell in the body expresses its own distinctive surface signature antigens so that it can recognise and link up with other cells of the same lineage and can also make itself known as “self” to immune cells guarding against foreign invaders. Thus, the neurons forming the dorsal pathway are closely related and distinct from those forming the ventral pathway. They appear to be particularly genetically vulnerable and are often damaged during development. Moreover, since their development is controlled by the same histocompatibility genes that set up their surface signatures [20], they are especially vulnerable to autoimmune attack.

## Magnocellular Impairment in Dyslexia

Thus, the visual magnocellular system plays a crucial role in directing attention, preventing blur from miniature eye movements and locking the eyes on the target. All of these are vital for reading, so the accumulating evidence that the M system is poorly developed in many dyslexics has special significance. Although the theory that a visual M cell deficit underlies visual problems in reading is still highly controversial [21], over 90 % of the studies performed over the last 10 years that have sought evidence of M impairment in dyslexics have found it in at least some of them.

## The Subcortical M System

Strictly speaking, as we have seen, visual M cells can be rigorously defined only in the subcortical visual system because only in the retina and LGN are they anatomically separated from P cells. Thereafter, the magno and parvo systems converge and interact strongly. Hence, the only way to be sure that deficits in dyslexics are confined to the M system is to use stimuli that are selectively processed by the subcortical M neurones in the retina and LGN. Skottun has made this point repeatedly. But even if we confine ourselves to the M system in the retina and LGN, the evidence of M impairment in dyslexics is strong.

## Contrast Sensitivity

Perception of low contrasts in the environment is mainly set by the properties of M ganglion cells in the retina. The simplest way of assessing their variability in individuals is to measure subjects’ sensitivity to the contrast of coarse gratings (spatial frequency <1 cycle per degree) flickered at high temporal frequencies of >10 Hz, since only M cells can respond to this combination. Since the first report [22], several studies have confirmed that the contrast sensitivity (CS) of many dyslexics is lower than that of controls but only at the low spatial and high temporal frequencies mediated by the M system [23–25]. The fact that CS at high spatial and low temporal frequencies was normal in these dyslexics confirms that the deficit is selective for the M system.

## M Cell Nonlinearity

One of the most characteristic features of M cells is their nonlinear character [10]. They fire transiently not only when a light is switched on in their receptive field centre but also when it is switched off (rectification), so that if a grating is moved across the field, they will fire at twice the temporal frequency of the grating. Since they are much more sensitive to low contrasts than parvo cells are, as contrast is increased from zero, they are the cells that begin to respond much earlier than the P cells. Since they signal at twice the frequency of the stimulus, this may be interpreted perceptually as the grating not only moving at twice its true rate but also as having twice the number of bars. This has been termed the “spatial frequency doubling effect”, and it is thought, therefore, to constitute a selective test of retinal magnocellular sensitivity [26]. The details of this dependence have been contested [27]; nevertheless, whether or not the grating appears twice as fine, it is accepted that as the contrast of such a flickering grating is increased, the threshold at which observers first have enough contrast to see the pattern is determined by their retinal M cells. Dyslexics have been consistently shown to require more contrast to see the gratings, confirming their M cell weakness [28, 29].

The same nonlinearity can be seen in the visual evoked potential recorded from the primary visual cortex. If an oscillating grating or chequerboard is used as a stimulus, the M cells fire twice each time a bar crosses their receptive field, i.e. they fire at the second harmonic of the stimulus frequency. Since parvo cells fire at the entry of the bar only and are inhibited by its exit, they mainly respond just to the fundamental rather than the second harmonic. So, by measuring the ratio of the second harmonic to the fundamental frequency in the evoked potential recorded over the occipital pole, we can measure the sensitivity of an individual’s M system relative to his P system. We have found that this ratio is much lower in dyslexics [30], and this has turned out to be a sensitive technique for detecting magnocellular impairments, even in individual dyslexics. Unfortunately, most M tests are not sensitive enough to identify weakness in individuals—they identify only the average difference between groups of dyslexics compared with controls.

## Visual Jitter

As mentioned earlier, the M cells help compensate for miniature eye movements—the essential microsaccades and drifts that prevent total pigment bleaching and preserve vision during fixations. When patterns imaged onto one part of the retina are jittered for several seconds by a small amount equivalent to the average size of these miniature eye movements, the M cells there adapt and so give a falsely low estimate of the amplitude of the motion. Hence, after the jitter is stopped, images on unadapted adjacent parts of the retina will now appear to jitter. The duration of this “jitter illusion” therefore gives a measure of the sensitivity of retinal M cells. The more sensitive they are, the more adapted they will become, and so the longer they will take to return to normal after the jitter stimulus, and the longer the subsequent jitter illusion will be [9]. We have recently shown that in adult dyslexics, this illusory percept is much shorter, and its duration correlates not only with other measures of M cell function but also with the degree of that individual’s reading retardation (Stein et al. The visual jitter illusion is briefer in dyslexics. In preparation).

M cells are responsible for timing visual events, so their sensitivity in individuals can also be assessed by various timing tests. For example, Lovegrove et al. [22] tested people’s ability to detect a discontinuity (a temporal “gap”) in the display of a low-contrast, low–spatial frequency grating and found that dyslexics needed a much longer gap than controls. When the frequency at which a light is flickered is increased above around 30 times per second, the M system can no longer respond fast enough, and the light appears to cease flickering. This is the “critical flicker fusion frequency”. Several studies [23, 31–34] have all shown that this frequency tends to be lower in dyslexics.

## Contrary Evidence

However, some studies have failed to confirm M impairments in dyslexics. On the basis of his study with Gross-Glen of 18 adult dyslexics [35], together with more than 30 reviews of others’ work, Skottun [36] has repeatedly criticized the hypothesis that dyslexics have specifically impaired M stream processing, even though he accepts that many dyslexics have visual problems. The reading of Gross-Glen’s sample of dyslexics was actually better than normal for their age but was behind that expected for their IQ. Although it was found that the dyslexics did have CS deficits when they were compared with good readers, these were not so marked at the low spatial frequencies that are expected to stimulate the magno system best. Instead, they were more impaired at higher frequencies. However, Gross-Glen et al. used very brief stimuli lasting only 17 or 34 ms (equivalent to temporal frequencies of 59 and 29 Hz, respectively)—frequencies that would preferentially stimulate the M system. Thus, Gross-Glen’s results actually confirmed others’ findings that if gratings are flickered at high temporal frequencies, dyslexics show lower CS than controls even at high spatial frequencies; hence, dyslexics often show M weakness.

Williams et al. [37] also failed to find any significant differences between dyslexics and controls at either low or high spatial frequencies. However, they studied only a small number of participants, and they chose to stimulate the M system by using a high-contrast grating at a temporal frequency of only 8 Hz. The P system is not completely silenced at this high contrast and rate, and it can still respond, so large differences would not be expected, especially with such a small number of participants.

Sperling et al. (2005) have suggested that dyslexics’ visual problems are the result not of a magnocellular impairment but of a failure to filter out “visual noise”. Clearly, the source of such noise is crucial. Probably the impaired M system in dyslexics spatially undersamples the visual world [38]. This would leave response gaps between retinal M cells, which would clearly add noise to any visual processing, just as Sperling et al. found.

Like Gross-Glen [35], Sperling et al. failed to find lower CS in dyslexics at the low spatial frequencies, which they expected of an M impairment. But they used stationary gratings, whereas for stationary gratings, CS is not mediated by the M system alone, even at low spatial frequencies [39]. Hence, their dyslexics’ surviving sensitivity to low spatial frequencies was probably mediated by the parvo system because this can signal even low–spatial frequency gratings if they are stationary.

A flickering light is equivalent to a very low–spatial frequency and high–temporal frequency stimulus, and almost all those who have measured dyslexics’ flicker sensitivity have found it to be slightly reduced [40].

In summary, however, the great majority of studies that have specifically looked for subcortical visual M cell deficits in dyslexics have shown that many of them do suffer from mild impairments, particularly at high temporal and low spatial frequencies.

## The Lateral Geniculate Nucleus

Further support for the idea of M cell impairment in dyslexics came from Galaburda and colleagues’ postmortem histological studies of dyslexic brains from the Orton dyslexia brain bank at Harvard University [41]. They found that the M layers in the LGN in the dyslexic brains were selectively impaired. Not only were the cells approximately 25 % smaller in the dyslexic brains than in the control brains, but also the M cells were not confined to their proper M layers; many had mismigrated into the adjacent konio and parvo layers of the LGN. However, only a very small number of dyslexic brains have been examined postmortem in this detail, so we must treat these results with caution.

## The Cerebellum

The cerebellum is the brain’s autopilot, responsible for automatising motor skills by building up internal models to mediate their accurate execution [42, 43]. As we have seen, it plays an important part in stabilising our visual world by predicting the image movements that will occur when we move our eyes, so that they can be subtracted from our conscious perception to keep the world stationary. Since accurate timing of the image movements and motor outflow is an essential requirement for this function, the cerebellum receives a rich input from the visual, auditory, proprioceptive and motor magnocellular systems [7, 44]. Furthermore, cells in many of the precerebellar nuclei that provide inputs into the cerebellum, together with the cerebellar Purkinje cells themselves, stain for the magnocellular-specific antigen CAT-301, which was described earlier. Thus, the cerebellum can be considered almost part of the magnocellular system. Accordingly, there is now a great deal of evidence that cerebellar function is also mildly impaired in dyslexia and related neurodevelopmental conditions, such as developmental coordination disorder (dyspraxia), attention-deficit/hyperactivity disorder (ADHD) and autism [45–47]. This provides yet further indirect evidence of magnocellular involvement in dyslexic problems.

## The Cortical Dorsal “Where” Pathway

As we have seen, this pathway is dominated by visual M input, and many of the cells stain for CAT-301. Abnormalities have been found in dyslexics in this pathway at every level from the prestriate visual motion area (MT/V5) via the PPC to the ultimate goal of both the M and P systems—the prefrontal cortex, where decisions are made about what to do about events perceived in the visual world [48].

## Random Dot Kinematograms

Ninety percent of the visual input into the motion-sensitive neurons in the middle temporal visual motion area (MT/V5) is provided by the M system, and only 10 % comes from other sources. A good way of assessing the sensitivity of these MT neurons in individuals is to measure their responses to visual motion in “random dot kinematograms” (RDKs). Clouds of dots moving in the same direction, “coherently”, are progressively diluted with noise dots moving in random directions until the subject can no longer detect any coherent motion in the display. This threshold therefore defines that individual’s motion (visual dorsal stream) sensitivity. Several researchers have shown that this is reduced in many dyslexic individuals [23, 49–53]. Other work has similarly shown reduced velocity discrimination [54–56] and elevated speed thresholds for motion-defined form [32, 57].

People with low motion sensitivity can still be adequate readers, however [58], so weak M function by no means predestines a child to reading failure. Other vulnerabilities must contribute to dyslexia as well. Nevertheless, individual differences in motion sensitivity explain over 25 % of the variance in reading ability in both bad and good readers [51]. In other words, individuals’ dorsal stream performance, dominated by M cell input, plays an important part in determining how well visual reading skills develop, and this is true of everybody, not just those diagnosed with dyslexia.

## Higher-Level Dorsal Stream Tasks

The PPC receives its main visual input from MT/V5; this input plays a crucial role in its functions of guiding visual attention and eye and limb movements [59]. Dyslexics have been found to be worse than good readers at cueing visual attention [60–62], visual search [63, 64], visual short-term “retain and compare” memory [65] and attentional grouping in the Ternus test [66]. These findings again suggest that their dorsal stream function is impaired.

But—as has been repeatedly pointed out by Skottun—because none of these tests stimulates the peripheral magnocellular system entirely selectively, these results do not by themselves prove that impaired magnocellular function is responsible [21]. Nevertheless, as the dorsal stream is dominated by M input, its contribution will be the most important. Moreover, many of the studies mentioned above incorporated control tests for parvo function, such as visual acuity and colour discrimination, and dyslexics usually proved to be as good or better at these. This suggests that their poor performance can be mainly attributed to M system weakness even in the presence of robust parvocellular function [67].

## Cross-Modal Attention

Dyslexics not only show slow deployment of visual attention but also show difficulty shifting their attention between sensory modalities—for instance, between vision and hearing—but it seems that such “sluggish attention shifting” [68] is worst when dyslexics shift their attention from the visual to the auditory modality, rather than vice versa [69]. Thus, dyslexics are not only slower but are also particularly slow when they have to attend to a visual stimulus and shift to an auditory one, as when reading. Since these shifts of attention have been shown to depend on the M system, this result provides further evidence in favour of M impairments in dyslexia.

Taken together, all of this evidence suggests that dyslexics’ poor dorsal stream performance can be mainly attributed to M system weakness even in the presence of robust parvocellular function [67].

## Eye Movement Control by the Dorsal Stream

Normally, the dorsal, M-dominated stream not only directs visual attention towards a target but then also directs the eyes towards it. Hence, numerous studies have found not only that the direction of visual attention is disturbed in dyslexics [70, 71] but also that their eye control during reading is poor. In particular, their saccades are shorter, their vergence is poorly controlled, their fixations are longer and they make more regressions to re-examine words they have already read [72–76].

However, it is strongly argued that these abnormalities are not a cause of reading problems but are the result of not understanding the text; hence, the person has to make longer fixations and more re-inspections of previous letters to try to decode words [72]. This is generally accepted, but it is probably not the full story. Poor eye control in dyslexics has also been demonstrated in several non-reading situations, e.g. measuring fixation stability inspecting non-text targets [77] and recording smooth pursuit of moving objects and saccadic control [78, 79]. These findings suggest that poor eye control may come first and may itself be a significant cause of reading problems.

In one study, however, although dyslexics did display abnormal eye movements, these were not significantly associated with worse dorsal stream function as measured by coherent motion detection, but the number of dyslexics studied was very small [80].

In conclusion, the majority of studies have found not only that the direction of visual attention is disturbed in dyslexia but also that this impairment correlates with measures of M sensitivity in the same subjects.

## Event-Related Potentials

Recording-averaged electroencephalographic (EEG) potentials in response to a moving, low-contrast visual target probably provide a more objective measure of cortical dorsal stream processing than psychophysical techniques. Of recent visual event-related potential (ERP) studies in dyslexics, the great majority have either confirmed [41] the original observation that dyslexics have weaker responses to moving, low-contrast targets than good readers (e.g. [81]) or they have found that dyslexics show slower, smaller and spatially abnormal visual attentional ERP responses in line with psychophysical results. Only one recent study with small numbers of subjects found no sensory or attentional abnormalities [82]. In a recent review, it was concluded by Schulte-Körne and Bruder [83] that there is now consistent evidence that dyslexics show magnocellular deficits, as shown by reduced visual evoked potentials in response to rapidly moving stimuli presented at low contrasts.

## Reading Age Matches

In the majority of studies discussed so far, dyslexics have been compared with chronological age–matched controls, and the assumption is that if they show impairment in a task, that impairment is likely to be a cause of their reading problems. But, as Bradley and Bryant [84] pointed out, the cause and effect might be the other way round. Poor reading might be the cause of their poor performance on the task; lack of reading experience may cause, rather than result from, poor visual motion sensitivity, for example. They introduced the reading age match as a way of getting round this problem, suggesting that dyslexics should be compared with younger good readers with the same reading age and hence the same reading experience. Then, they argued, if the dyslexics still showed worse motion sensitivity than the reading age–matched controls, the dyslexics’ poorer sensitivity must have caused their worse reading for their age. However, if the dyslexics had the same visual motion sensitivity as the reading age–matched children, one still would not know which was the cause or effect. Both groups’ sensitivity might be determining their reading, or their reading experience might be governing their visual motion sensitivity. However, the latter seems pretty implausible, since motion sensitivity is a much more basic visual function than reading; nevertheless, exactly this has recently been claimed by Olulade et al. [85].

They studied only 12 dyslexics and 12 younger good readers, matched with the older dyslexics on reading age, and with an unusually large number of girls in both groups. They found that both groups had equivalent visual motion activity in V5 on functional magnetic resonance imaging (fMRI), and they argued that this meant that their lack of reading experience caused them to have visual motion sensitivity as low as that of the younger controls. However, other researchers using larger numbers have shown that reading age–matched children without visual reading problems usually have better sensitivity to dynamic visual stimuli [86]. But, even if their motion sensitivity was the same, this shows no more than that if you are 10 years old and you have the motion sensitivity of only a 7-year old, then you cannot read better than a 7-year-old. It is probable that the poor motion sensitivity of Olulade’s dyslexics helped to cause their poor reading, rather than vice versa.

In fact, in this situation, only intervention or cohort studies are able to demonstrate cause and effect. If increasing visual motion sensitivity during development precedes and predicts reading progress, then it is likely to be playing a causal role. Likewise, if an intervention such as coloured filters (see later) accelerates motion sensitivity and reading progress, then its effect on visual processing is likely to be causing the reading improvement [87]. Oululade et al. therefore used the Lindamood dyslexia training programme as an intervention to remediate the dyslexic children’s reading, and they found that this was associated with improved visual motion sensitivity. This is a so-called phonological programme, so the improvement in visual motion sensitivity was treated as an irrelevant side effect. But, actually, the Lindamood programme is very visual; it is called “Seeing Stars” for a good reason, namely that the programme teaches symbol imagery—“the ability to visualize the identity, number, and sequence of sounds and letters within words”—to quote Patricia Lindamood. Therefore, it probably improved these skills by training the magnocellular system; hence, these children’s improved motion sensitivity was probably not an epiphenomenon, but was causal.

## Visual Treatments

The main purpose of trying to understand the mechanisms causing dyslexics to have reading problems is to develop rational means of helping them. Although evidence that a particular technique works does not usually prove that the theory underlying it is correct, it provides circumstantial evidence in its favour. Although there is a plethora of claims about visual treatments that help dyslexics learn to read better, very few have been rigorously designed or properly controlled for placebo effects. The very fact that somebody is taking notice of their reading problems is often sufficient for children to try harder, to focus their attention more effectively and to feel better about themselves. So their reading may improve because of these strong Hawthorne effects even if the treatment has no specific effect at all. These effects should not be denigrated, however. Whatever their cause, any improvements should be welcomed. However they do not justify charging large sums for the treatment, as is often the case—and, to be plausible, they need to have a defensible rationale.

Here, therefore, I will only consider a few techniques that have a rationale that is relevant to the magnocellular theory, and that have been subjected to appropriate controls. In 1985, we published the results of a double-blind, randomised, controlled trial of monocular occlusion during reading [88]. We studied children with significant reading difficulties who also had unstable binocular control, measured using a standard orthoptic test. Using random selection, we gave them either spectacles with the left lens occluded with opaque tape or placebo clear plano spectacles. Those receiving the occlusion were more likely to achieve stable binocular control. If they did so, they improved their reading much more than those receiving placebo who did not achieve stable control (*p* < 0.005). In 2000, we repeated this study, using pale yellow spectacles as the placebo, and obtained similar results [89]. We argued that dyslexics’ binocular instability was likely to be due to a significant visual magnocellular deficit, and that occluding the left eye when reading helped the ocular motor control system to overcome this deficit to achieve more stable fixation.

## Yellow Filters

We noted that the placebo response to pale yellow filters in 2000 was considerably greater than the placebo response to plano clear lenses in 1985. We had also found that we could improve binocular amblyopia in some children by using deep yellow filters [90]. We argued, therefore, that these effects occurred because yellow filters actually stimulate the magnocellular system specifically. This is because although the M ganglion cells do not contribute to conscious colour vision, they receive mainly from the long wavelength (red) and medium wavelength (green) cones that are activated maximally by yellow light. The yellow filters reduce the total amount of light entering the eye; hence, they cause pupillary dilation and actually increase the amount of yellow light falling on the retina. Therefore, we tested whether deep yellow filters designed to maximally activate M cells might be even more effective in improving M cell function and reading in children with visual reading difficulties.

In a subsequent, double-blind, randomised, controlled trial, we showed that this was indeed the case [87]. Those who received the yellow filters improved their M responses, as indexed by their sensitivity to visual motion in RDKs, and this improvement was accompanied by improved single word reading (*p* < 0.05). We have since confirmed that the yellow filters actually do increase the amount of long-wavelength light falling on the retina, hence stimulating M cells more, because the pupil dilates.

## Blue Filters

We were using blue filters as active controls for the effects of the yellow filters. But, to our surprise, we found that some children actually benefited more from wearing the blue filters than from the yellow filters. Recently, a new class of intrinsically photosensitive retinal ganglion cells (IPRGCs) has been discovered. They contain the blue-sensitive pigment melanopsin [91]. These cells project not to the retino-geniculate conscious visual system but to the suprachiasmatic nucleus (SCN) in the hypothalamus. The latter contains the body’s internal clock, which controls our diurnal rhythms. Thus, the function of its input from the melanopsin-containing retinal ganglion cells is not to mediate conscious vision but to synchronise the SCN to seasonally varying day length. We wake up earlier in the summer because these ganglion cells signal the amount of blue light entering the eye, which is maximal in the morning, and the SCN then arouses us.

For this arousal, the SCN appears to activate the noradrenergic locus coeruleus to specifically facilitate the magnocellular layers of the thalamic LGN and the dorsal “where” visual pathway and its magnocells. Hence, we suggest that when we give children blue filters, we facilitate this dorsal visual route and help the children to focus their attention and eye movements more reliably. Accordingly, we have recently shown in another randomised, controlled trial that giving children with visual reading problems blue filters increases their reading accuracy significantly more than giving them yellow filters does.

Improving the synchronisation of diurnal rhythms has other benefits as well. Many children with visual reading difficulties have disturbed sleep patterns (reference). Their parents are often surprised that the blue filters seem to improve their child’s sleeping. Likewise, many such children complain of headaches when they try to read. Migraine headaches are known to be accompanied by disturbed sleep rhythms. Hence, we now have many anecdotal reports that successful treatment of reading difficulties with blue filters is accompanied by fewer headaches, and we are now following this up more systematically.

## Other Colours

However, there are now many commercial companies selling a wide range of coloured filters for improving reading problems, with the claim that each person needs an individually prescribed colour for best effect; hence, they argue that using only yellow and blue filters would fail to meet individual requirements. Wilkins et al. [92] studied 68 children who viewed text illuminated by coloured light in an apparatus that allowed separate manipulation of the hue (colour) and saturation (depth of colour) at constant luminance. However, 31 of the children (46 %) dropped out of the study altogether, presumably because the glasses did not help them. Of those who did complete the study, slightly more reported a reduction in symptoms of eyestrain and headache when the light had a chromaticity within a limited range, which was different for each individual.

A pair of plastic spectacle lenses was then dyed so as to provide the appropriate chromaticity under standard white fluorescent light for each child. A placebo pair was also made that appeared to be a similar colour but had a chromaticity outside the range that the child had reported to improve their visual symptoms. Each pair was worn for 1 month. The children kept diaries recording their symptoms, but only 36 (53 %) were completed. Symptoms were indeed slightly less frequent on days when the correct lenses had been worn, but the difference was small. Interestingly, the chromaticities of the effective filters mostly clustered around yellow or blue, and there was no evidence that simple yellow and blue would not have been equally or more efficacious [92].

Thus, the evidence for requiring a large range of filter colours is slight; the individual colours that are chosen usually cluster around yellow and blue, and if the M theory outlined here is correct, only yellow and blue should suffice. We have now compared our Oxford blue and yellow filters with one company’s much wider range of colours. The Oxford filters actually achieved superior results [93].

In summary, there are now a fair number of clinical trials showing that visual treatments derived from the visual magnocellular theory of reading problems have produced worthwhile improvements in children’s reading. These will remain controversial until many more, larger, properly controlled trials have been carried out. But they are at least consistent with the M theory.

## Causes of M Cell Impairment

Of course, the really interesting question is why dyslexics may have impaired development of these magnocellular systems at all. There are three interacting factors that I will consider here: genetic, immunological and nutritional.

## Genetics

In his book *Hereditary Genius*, Galton (1869) proposed that a “system of arranged marriages between men of distinction and women of wealth should eventually produce a superior race”. He coined the term “eugenics” in 1883 to promote these arrangements and continued to expound their benefits until his death in 1911. Henry Maudsley, who rebuilt the Bedlam (now Maudsley) Hospital in London, using his own money, initially supported these ideas. But later, his psychiatric experience led him to oppose them strongly. As the foremost psychiatrist of his day, he observed that most of the great creative and successful families in Victorian England sheltered relatives who had “mental” problems. He judged it dangerously simplistic to believe that mental disease was entirely hereditary, and he feared that eliminating the genes that influence mental health might also eliminate the creativity and imagination that he saw so strongly in these families. He had worked out intuitively that the genes that help to cause conditions such as dyslexia, depression and schizophrenia would not be as common as they are unless they also contributed to the talents that made these families so successful. But not until the 1930s did eugenic theories come under serious attack when the German Nazis began to exploit them to support their extermination of Jews, gypsies and homosexuals.

I begin this section on this note of caution to urge us to ponder why we should study the genetic basis of dyslexia at all. We certainly should not be trying to “root out dyslexia genes”, because thereby we might eliminate our chances of benefiting from future highly talented dyslexics—the likes of Leonardo da Vinci, Albert Einstein or Winston Churchill. If pure curiosity to unravel the genetic basis of dyslexia were to have that effect, then we should vigorously oppose it.

However, understanding how genes influence reading ability should have far more positive effects. Perhaps the most important gain that has already accrued from the uncontested demonstration that dyslexia has a strong genetic component is that this proves absolutely that dyslexia is a real neurological condition, and not a euphemism to hide middle-class children’s laziness or stupidity. Demonstrating that it has a genetic basis makes it impossible to maintain that it is “purely psychological”; rather, it shows that dyslexia has a clear biological reality. Knowing that his/her dyslexia is a respectable neurological diagnosis, and not another word for laziness or stupidity, can transform a child’s self-image. From losing all self-esteem because he could not keep up with his peers, giving him the diagnosis of dyslexia returns his self-respect and hope, and it gives him the confidence to exploit talents that have often been hidden by his shame.

Writing was only invented approximately 4,000 years ago, so literacy itself is a cultural development and is not likely to be under direct genetic control. Instead, reading and writing must have piggybacked on more fundamental sensory and motor processing functions that originally evolved for other purposes. These develop under the combined influence of genes and the environment. Hence, another strong reason for studying the genetics of dyslexia is to help elucidate the mechanisms by which the development of the human nervous system contributes to reading skills. Then we will have a better chance of understanding not only more about how these basic neural processes work but also how they come to mediate reading.

One great advantage of applying genetic techniques to the study of the development of reading skills is that reading is much easier to measure precisely than many other higher functions, such as emotion, motivation or delusional thinking. As a result, unlike the 100 or so genes of small effect that have been implicated in schizophrenia [94], fewer than a dozen genes with much larger effects have been associated with dyslexia, and their role in reading is steadily being unravelled [95].

We have taken advantage of the large number of children and families with reading problems that we have seen in our clinics around Oxford to carry out whole-genome quantitative trait linkage (QTL) studies. We collected data on nearly 500 Oxford families and replicated many of our findings in a data set of 200 Colorado families provided by Richard Olsen [96, 97]. I shall just discuss two new genes that these analyses revealed.

## KIAA0319

The first of these is a gene on the short arm of chromosome 6 in amongst the major histocompatibility complex (MHC)—a gene named KIAA0319 [98]. This appears to be underexpressed in dyslexia, and the protein it encodes is now known to be a partly extracellular cell-to-cell signalling molecule. In the normal course of the early development of the cerebral cortex, neurones born in the ventricular zone migrate up the radial glia to their correct positions on the surface to form the six layers of the cerebral cortex. If KIAA0319 is completely switched off by local electroporation of a specific inhibitory RNA interference in the rat embryo brain, the cells fail to migrate at all and remain clustered around the ventricle [98].

Although the gene appears only to be underexpressed in dyslexia, rather than completely knocked out, reduced production of the cell-to-cell signal could explain the mismigration of M cells that is found in dyslexic brains postmortem. These anomalies have been seen in the magnocellular layers of the visual LGN, in the magnocellular portion of the left auditory medial geniculate nucleus and in the form of outgrowths of neurones (ectopias) in the cerebral cortex. The latter particularly seem to affect large neurones contributing to the language network in the left hemisphere.

At least two other genes involved in the control of neural migration early in the development of the brain have also been associated with dyslexia [99]. Unravelling the precise function of these genes promises to revolutionise our understanding of neurodevelopmental disorders and with it, hopefully, our ability to treat them successfully. This applies not only to dyslexia but also to the whole gamut of neurodevelopmental conditions that overlap with it both genetically and phenotypically, such as developmental dysphasia (specific language impairment), dyscalculia, developmental dyspraxia, ADHD, autism, bipolar disorder and schizophrenia.

## Autoimmunity

Because their development is under the control of the MHC gene complex [20], with the gene KIAA0319 in their midst, one way of identifying magnocells throughout the nervous system is to stain them for their characteristic surface antigen with antibodies such as CAT-301. Unfortunately, magnocells, which are so vulnerable in other ways, also seem to be particularly vulnerable to antibody attack. Antineuronal antibodies are found in the blood in many general autoimmune conditions, such as systemic lupus erythematosus (SLE). Ectopias similar to those seen in dyslexic brains are found routinely in the brains of BXSB mice, a strain of “autoimmune” mouse that has been bred as an animal model of lupus. So it is not surprising to find a very high incidence of dyslexia and other neurodevelopmental conditions in the children of mothers with lupus [100]. Interestingly, also, dyslexic children and their families consistently report a higher prevalence of immunological problems—not only lupus, which is rare, but also much commoner conditions such as eczema, asthma and allergies [101].

## Dyslexic Mice

Although most antibodies circulating in a mother’s blood fail to cross the placenta to affect her foetus, some can cross over and even reach the foetal brain. We therefore decided to see whether mothers with dyslexic children showed any signs of circulating anti-magnocellular antibodies in their blood. We took serum from mothers who had had two or more dyslexic children and injected it into the uteri of pregnant mice. We then tested the offspring of those mice for behavioural abnormalities and looked for anomalies in cerebellar metabolism by magnetic resonance spectroscopy (MRS) and in cerebellar antibody binding. We found that these young mice did indeed show deficits in motor coordination tests. These abnormalities were associated with antibodies binding to the pup’s cerebellar Purkinje cells, and their severity correlated with MRS indices of impaired cerebellar metabolism [102].

Taken together, therefore, these findings about the association between autoimmunity, magnocellular development and dyslexia provide further support for the hypothesis that a specific magnocellular impairment may underlie the manifold symptomatology of dyslexia.

## Nutrition: Omega-3 Fish Oils

Another chromosomal site that showed very strong linkage to reading difficulties in our Oxford and Colorado samples of dyslexic families was on chromosome 18 (18p11.2) [103]. This site is very close to the melanocortin receptor 5 gene (MCR5). But this receptor is not strongly expressed in the brain. Instead, it is probably involved in appetite control—in particular, affecting the metabolism of omega-3 essential fatty acids. The same site (18p11.2) has been implicated in susceptibility to bipolar depression [104]. There is evidence that both dyslexia and bipolar depression can be ameliorated by administering omega-3 supplements, as we shall see.

We are therefore particularly interested in the possible role of this gene in the metabolism of omega-3 long-chain polyunsaturated fatty acids (LCPUFA) derived from fish oils. A single LCPUFA, docosahexaenoic acid (DHA), makes up 20 % of all neuronal membranes [105]. It has just the right properties to contribute flexibility and the correct electrostatic profile to the membrane. As such, it has been conserved in eukaryotic membranes throughout evolution since the Cambrian explosion 600 million years ago. There are cogent reasons for believing that because we evolved near water, our ready access to this molecule from eating fish explains how our brains came to be so much larger in relation to our body size than is the case in most other animals. DHA seems to be particularly important for proper magnocellular neuronal function because it is kinky and thus prevents the lipid molecules in the membrane from packing together too tightly. This confers flexibility in the membrane to allow ionic channels to open and close very fast.

But DHA is continuously leached out of the membrane by phospholipases because it also forms the basis of many prostaglandin, leukotriene and interleukin signalling molecules. Likewise, another LCPUFA, eicosapentaenoic acid (EPA), is the substrate for other eicosanoid prostaglandins, leukotrienes and resolvins. They all tend to be anti-inflammatory [106]. Magnocells are thought to be particularly vulnerable to lack of these LCPUFAs, which are derived from oily fish.

Our modern Western diet is dreadful, however, because we consume far too much salt, sugar and omega-6 fats—the dangerous “3 S’s”—and we eat far too little oily fish, fat-soluble vitamins or minerals; 75 % of teenage boys eat no fish at all. Hence, a high proportion of the population, particularly from deprived households, is dangerously deficient in these essential nutrients. In randomised, controlled trials, we found that simply giving deprived children supplement capsules containing EPA and DHA from oily fish could improve their visual magnocellular function. Hence, the children’s ability to focus their attention improved, and their reading benefited greatly [107].

We also observed that the children we were studying appeared calmer and less aggressive in the playground. A probation officer, Bernard Gesch, therefore tested whether improving nutrition with supplements of omega-3 s, minerals and vitamins could improve their behaviour. He designed a double-blind, randomised, controlled trial to compare active supplements with placebo in nearly 300 young male offenders in a tough young offenders’ institute. The active supplements reduced these prisoners’ rate of offending by over one third—“peace on a plate” [108]. We have recently completed a larger study that reached the same conclusion (Gesch & Stein, in preparation). Thus, simply improving these young lads’ diets can help them to control themselves better and therefore behave less antisocially. If such a simple and cheap solution really is that powerful, it will have profound implications.

## Conclusion

The genetic, developmental, nutritional, neuroanatomical, physiological and psychophysiological evidence that I have reviewed here all supports the view that dyslexics’ reading problems may be due to mild, but pervasive, impaired development of the visual magnocellular system throughout the brain. However, definitive proof of this will only come when we have fully worked out how genetic and environmental influences determine the development and subsequent function of these cells.
